# A novel EGFR exon 21 indel mutation in lung adenocarcinoma and response to dacomitinib: A case report

**DOI:** 10.1097/MD.0000000000030269

**Published:** 2022-08-26

**Authors:** Tao Zhou, Qiang Xiong, Chen Hong, Qian Wang, Wenxian Wang, Chunwei Xu, Jing Cai

**Affiliations:** a Department of Oncology, The Second Affiliated Hospital of Nanchang University, Nanchang, China; b Jiangxi Key Laboratory of Molecular Center, Nanchang, China; c Department of Respiratory Medicine, Affiliated Hospital of Nanjing University of Chinese Medicine, Jiangsu Province Hospital of Chinese Medicine, Nanjing, China; d Department of Medical Oncology, The Cancer Hospital of the University of Chinese Academy of Sciences (Zhejiang Cancer Hospital), Hangzhou, China; e Department of Respiratory Medicine, Jinling Hospital, Nanjing University School of Medicine, Nanjing, China.

**Keywords:** case report, dacomitinib, EGFR mutation, indel, lung adenocarcinoma

## Abstract

Uncommon epidermal growth factor receptor (EGFR) mutations are increasingly being identified in non-small cell lung cancer. Insertion and deletion mutations have been detected in exons 18, 19, and 20, but not in exon 21. In patients with uncommon mutations, the second-generation EGFR tyrosine kinase inhibitor afatinib has shown good efficacy, whereas that of dacomitinib, another second-generation EGFR-tyrosine kinase inhibitor, remains unknown. Here, we reported a patient with a novel EGFR21 exon insertion-deletion (indel) mutation and demonstrated the efficacy of dacomitinib.

**Patient concerns:**

A 59-year-old nonsmoking Chinese male was admitted to the hospital with lung cancer after a chest computed tomography for coughing and sputum. The patient’s condition progressed after multiple treatments including surgery, chemotherapy, and radiotherapy.

**Diagnosis:**

The patient had clinical manifestations of cough and sputum and was pathologically confirmed to have T2bN1M0 (stage IIB) lung adenocarcinoma according to the seventh edition of tumor-node-metastasis staging. The patient underwent a second operation after detection of recurrence, and postoperative pathology confirmed adenocarcinoma of the lung. The patient progressed again after surgery, and the tumor-node-metastasis stage was changed to T4N0M1a (stage IVA) before treatment with dacomitinib.

**Interventions:**

After detection of the EGFR exon 21 indel mutation, the patient began treatment with dacomitinib (45 mg once a day) on March 12, 2021.

**Outcomes:**

After 1 month of targeted therapy, the patient showed a partial response to dacomitinib. As of March 19, 2022, his condition remained stable and he continued to receive dacomitinib. Progression-free survival reached 12.4 months. The patient experienced mild adverse reactions of pruritus during the use of dacomitinib, but recovered after drug treatment.

**Lesson:**

We reported a novel EGFR exon 21 indel mutation in a lung adenocarcinoma patient. Dacomitinib showed efficacy in the treatment of a patient with this mutation, suggesting that its efficacy in patients with uncommon mutations should be explored further. The next-generation sequencing is recommended as a guiding tool for the treatment of advanced non-small cell lung cancer.

## 1. Introduction

More than 600 types of epidermal growth factor receptor (EGFR) mutations are described in the Catalogue of Somatic Mutations in Cancer (COSMIC). In non-small cell lung cancer (NSCLC), the most common EGFR mutations are exon 19 deletion and exon 21 L858R point mutations. These mutations are highly sensitive to EGFR tyrosine kinase inhibitors (EGFR-TKIs). Rare mutations are defined as mutations other than EGFR 19del and L858R and account for only 10% to 20% of all EGFR mutations.^[1]^ The study of these rare mutations has progressed at a slow rate mainly because of tumor heterogeneity. Rare mutations are less sensitive to EGFR-TKIs. Afatinib, a second-generation EGFR-TKI, was previously shown to be effective in NSCLC patients.^[2]^ However, the efficacy of another second-generation EGFR-TKI, dacomitinib, in the treatment of rare EGFR mutations has not been demonstrated. Here, we identified, for the first time, an EGFR exon 21 insertion and deletion mutation. Previously, insertion-deletion (indel) mutations were only found in EGFR exons 18, 19, and 20, and mutations in exon 21 were mainly point mutations. In addition, we described a positive response to dacomitinib in a patient with a rare mutation.

## 2. Case report

**Figure 1.**
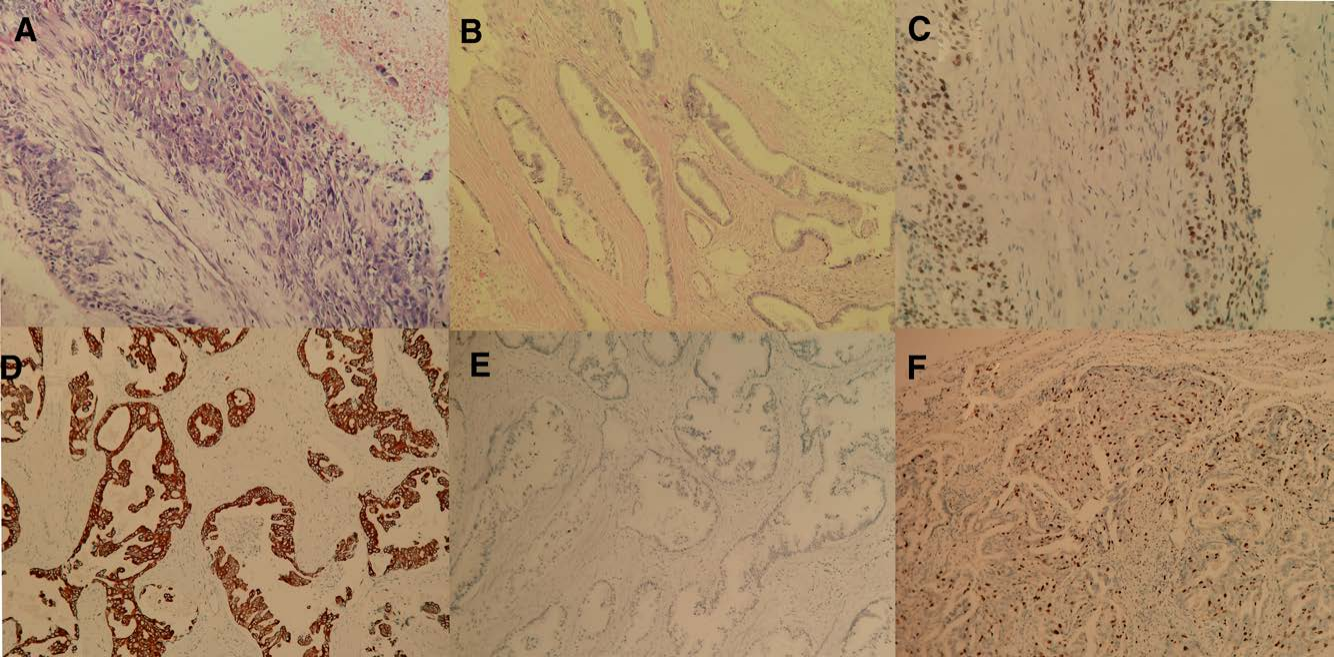
(A and B) Surgical excision of a lung tumor showed a lung adenocarcinoma (hematoxylin-eosin (HE) staining ×400). (C) Immunohistochemistry (IHC) revealed that the lung tumor cells were partially positive for P40 (×400). (D) IHC analysis revealed that the lung tumor cells were positive for CK7 (×400). (E) IHC analysis revealed that the lung tumor cells were negative for TTF-1 (×200). (F) IHC analysis revealed that the lung tumor Ki-67 index was 20% (×200). TTF-1 = thyroid transcription factor-1.

**Figure 2.**
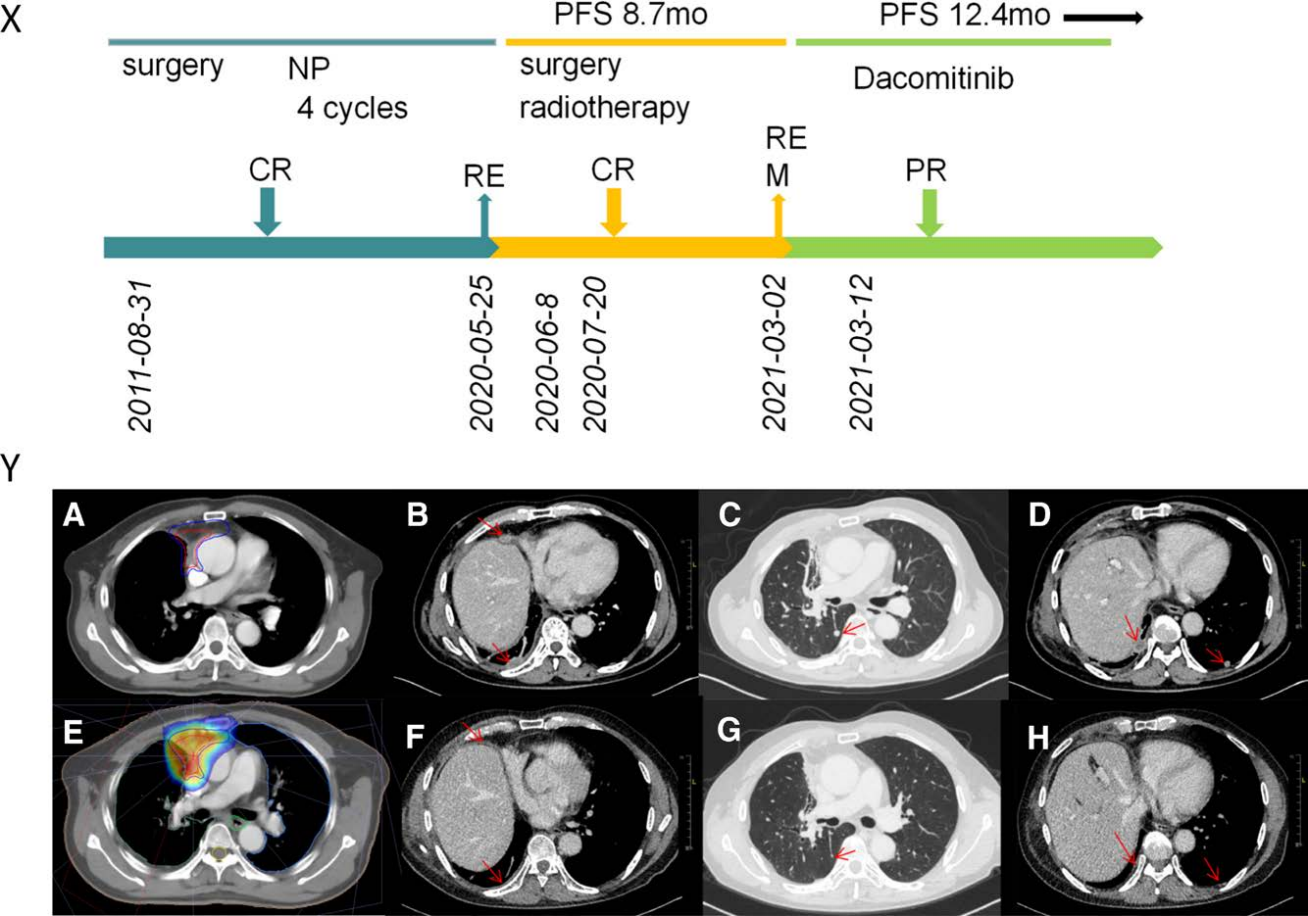
(X) Review of the treatment process (from August 2011 to December 2021). Treatment process and evaluation of the patient. CR = complete response, M = metastasis, mo = months, NP = vinorelbine, cisplatin, PFS = progression-free survival, PR = partial response, RE = recurrence. (Y) (A) The red lines represent GTV, including the tumor bed. The blue lines represent CTV, including the tumor bed and areas where the tumor may be involved. (E) Dose distribution map of radiotherapy. (B–D) Computed tomography scan before dacomitinib therapy. (F–H) Computed tomography scan of the chest showing a partial response after 1 month of dacomitinib treatment. CTV = clinical target volume, GTV = gross tumor volume.

In August 2011, a 59-year-old Chinese man presented to our hospital with cough and sputum production. Chest computed tomography (CT) showed a mass shadow and enlarged right hilar lymph nodes in the upper lobe of the right lung. Brain-enhanced magnetic resonance and whole-body bone imaging showed no abnormalities. The patient was diagnosed with T2bN1M0 (stage IIB) lung cancer according to the seventh edition of tumor-node-metastasis staging. The patient underwent radical resection of the upper right lung tumor on August 31, 2011, and the postoperative pathological diagnosis was lung adenocarcinoma. He received postoperative chemotherapy with 4 cycles of the vinorelbine plus cisplatin regimen. The patient remained in stable condition from September 2011 to April 2020. In May 2020, the patient presented to our hospital again with blood in sputum. On May 25, 2020, chest CT showed a right middle lobe and adjacent mediastinum occupying space. He underwent CT-guided percutaneous lung puncture and received a pathological diagnosis of lung adenocarcinoma. Thoracoscopic right lobectomy was performed on June 8, 2020. Pathological examination of postoperative specimens confirmed the diagnosis of lung adenocarcinoma (Fig. 1A and B). The tumor involved the thymus and striated muscle tissue. Immunohistochemistry showed that the tumor was partially positive for P40 (Fig. 1C), positive for CK7 (Fig. 1D), and negative for thyroid transcription factor-1 (Fig. 1E). The Ki-67 score was 20% (Fig. 1F). Because the tumor was in close proximity to the heart and ascending aorta, it was not completely removed during surgery. Postoperative radiotherapy began on July 20, 2020. The clinical target volume included the whole tumor bed and areas of possible tumor involvement and received a dose of 6000 cGy/30F, and the gross tumor volume, which included the tumor bed, received a dose of 6000 cGy/30F (Fig. 2Y [A, E]). A pulmonary metastasis was detected by chest CT in March 2021 (Fig. 2Y [B, C, D]) and the tumor-node-metastasis stage was changed to T4N0M1a (stage IVA). A deoxyribonucleic acid sequencing study of the patient’s tumor tissues detected single nucleotide mutations and indel mutations in exonic regions of 457 genes. An EGFR exon 21 p.L858_A859delinsRS indel mutation was detected, and the mutation abundance was 88.74%. A search of 4 databases, COSMIC, TCGA, ClinVar, and 1000 Genomes, did not yield information on the detected mutations. These genetic data have not been shared with the relevant archives. Dacomitinib inhibits human epidermal growth factor receptor signaling efficiently and is associated with superior progression-free survival and overall survival in patients with EGFR L858R point mutation.^[3]^ The mutation detected in the present study and the sensitive mutation L858R both had a change in the amino acid at the same site. Therefore, on March 12, 2021, the patient was started on oral dacomitinib (45 mg/day) therapy. After 1 month of treatment, the patient’s chest CT showed remarkable shrinkage of the tumor size (Fig. 2Y [F, G, H]). According to The Response Evaluation Criteria in Solid Tumors (RECIST1.1), the patient was considered to have a partial response to dacomitinib.^[4]^ The patient experienced only mild adverse reactions of pruritus during treatment with dacomitinib. The patient has achieved partial remission and progression-free survival for >12.4 months (Fig. 2X). His condition is still stable and he continues to be treated with dacomitinib.

Written informed consent was obtained from the patient for publication of this case report and accompanying images. This is a retrospective case report and institutional approval was not needed.

## 3. Discussion

There are currently several targeted drugs for patients with common sensitive mutations and a few rare mutations. For advanced lung cancer patients with sensitive gene mutations, targeted therapy is the most effective option.^[5,6]^ However, the sensitivity or resistance of many rare genetic mutations to targeted drugs is unknown. Several uncommon mutations in EGFR exon 21 have been identified, including L861Q, L833V, and H835L. These are point mutations, and afatinib shows only partial efficacy in patients with these mutations.^[7–9]^ EGFR exon 19 indel mutation and exon 20 insertion mutation have been investigated extensively, and the efficacy of current targeted therapy against EGFR 20 insertion mutation is not optimal.^[10,11]^ No mutations involving base insertion deletions have been identified in EGFR exon 21. The efficacy of the second-generation EGFR-TKI dacomitinib in patients with uncommon mutations has not been demonstrated conclusively. To the best of our knowledge, this is the first report of a patient with an EGFR exon 21 indel mutation. A search of 4 gene databases (COSMIC, TCGA, ClinVar, and 1000 Genomes) found no reports on this gene mutation. In the present case, disease progression occurred despite various treatments including surgery, radiotherapy, and chemotherapy whereas dacomitinib showed excellent antitumor effects.

Dacomitinib is a pan-ErbB inhibitor that binds to a cysteine residue in the adenosine triphosphate pocket and inhibits EGFR irreversibly.^[12]^ The irreversible inhibitory effect of dacomitinib on EGFR was also established in vitro. Dilution of the dacomitinib and ErbB enzyme combination to less than half of its maximum inhibitory concentration suppressed recovery of enzymatic activity. After removal of the dacomitinib-containing medium, no downstream phosphorylation was detected.^[13]^ In the latest treatment guidelines, dacomitinib is used in patients with NSCLC with EGFR exon 19 deletion and L858R point mutation. Afatinib has shown good efficacy in patients with uncommon mutations because of its broad and irreversible inhibition of the EGFR and human epidermal growth factor receptor families.^[2,14]^ Dacomitinib is also used for the treatment of rare mutations (e.g., exon 18 G719C, exon 20 S768I).^[15]^ The efficacy of dacomitinib in patients with osimertinib resistance caused by EGFR L718Q mutation was demonstrated previously.^[16]^ In vitro experiments support this idea.^[17]^ However, other reports have come to the opposite conclusion and do not consider the former conclusion correct.^[18]^ Therefore, the use of dacomitinib in patients with rare mutations remains controversial, and there is no conclusive evidence supporting the efficacy of dacomitinib in patients with uncommon EGFR mutations.

**Figure 3.**
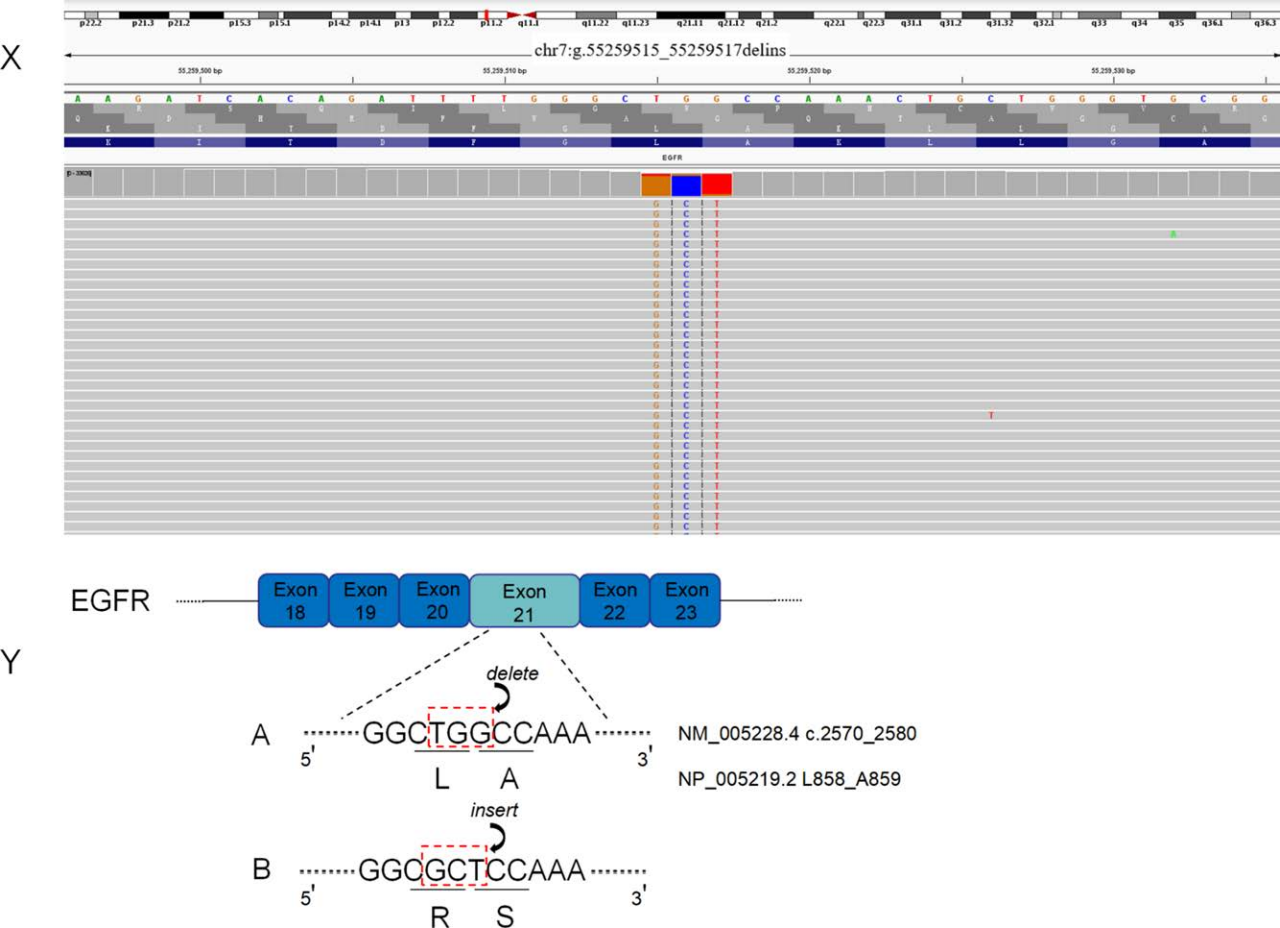
(X) Integrative Genomics Viewer snapshot of p.L858_A859delinsRS. (Y) Schematic diagram of insertion-deletion mutation in EGFR exon 21. A and B show the sequence of bases 2570 to 2580 of EGFR exon 21 cDNA. The transcript number is NM_005228.4. A is the sequence before the base deletion. B is the sequence of bases after insertion. Amino acids 858 and 859 are known according to NP_005219.2 A = alanine, L = leucine, R = arginine, S = serine.

We reported an uncommon mutation due to base insertion and deletion, resulting in the substitution of leucine at position 859 and alanine at position 859 of EGFR exon 21 by arginine and serine (Fig. 3). This may be similar to the EGFR L858R point mutation, which stabilizes the activation loop and increases the tyrosine kinase activity.^[19]^ This explains why dacomitinib is effective in such uncommon insertion and deletion mutations. However, during targeted therapy, the development of resistance to targeted medications is unavoidable.^[20]^ The prevailing view is that there are two resistance pathways. One is that EGFR produces a second mutation, which alters the site of drug action on EGFR, thereby preventing interaction with targeted therapy.^[21,22]^ In vitro studies show that dacomitinib causes T790M or C797S secondary mutations in pro-B-cell lines with EGFR19 deletion, L858R, or G719A mutations.^[23]^ In a phase 2 trial of dacomitinib as first-line treatment (ARCHER 1017), second EGFR mutations were detected in the blood of 15 patients, and 8 (53%) patients had T790M mutations.^[24]^ The second pathway is the activation of a bypass independently of EGFR, such as the activation of other receptor tyrosine kinases or the modulation of downstream signaling components.^[25]^ The patient presented in this study has not developed resistance to targeted therapy to date, and the follow-up is also worthy of attention. The development of resistance to targeted therapy in patients with uncommon mutations needs to be investigated in the future. Next generation sequencing (NGS) can identify a large number of unknown genes, which may benefit patients receiving targeted therapy. We also recommend NGS as one of the most important tests for advanced NSCLC.

In summary, we identified a new EGFR exon 21 indel mutation in a patient with lung adenocarcinoma. The patient achieved a significant and durable antitumor response after receiving dacomitinib targeted therapy. The present case thus supports the use of targeted therapy in patients with advanced lung cancer with rare EGFR mutations. The efficacy of dacomitinib in patients with other uncommon mutations will be the focus of future studies. Moreover, the NGS is recommended as a guiding tool for the treatment of advanced NSCLC. Because NGS can detect more known and unknown genetic mutations, these genes may be target genes or resistance genes for targeted therapeutic drugs.

## Author contributions

**Funding acquisition:** Jing Cai.

**Investigation:** Chen Hong.

**Project administration:** Qiang Xiong.

**Resources:** Chunwei Xu.

**Software:** Qian Wang,

**Supervision:** Jing Cai.

**Visualization:** Wenxian Wang.

**Writing the original draft:** Tao Zhou.

**Writing the review and editing:** Jing Cai.

**Conceptualization:** Chunwei Xu, Qian Wang.

**Funding acquisition:** Jing Cai.

**Software:** Wenxian Wang.

**Supervision:** Qiang Xiong.

**Writing – original draft:** Chen Hong, Tao Zhou.

**Writing – review & editing:** Chen Hong, Tao Zhou.
